# Physicochemical Attributes, Volatile Profile and Sensory Quality of Organic Crimson Crisp Apples during On-Tree Maturation

**DOI:** 10.3390/foods12071425

**Published:** 2023-03-27

**Authors:** Niklas Pontesegger, Thomas Rühmer, Barbara Siegmund

**Affiliations:** 1 Institute of Analytical Chemistry and Food Chemistry, Graz University of Technology, Stremayrgasse 9/II, A-8010 Graz, Austria; 2 Research Centre for Fruit Growing & Viticulture Haidegg, Ragnitzstraße 193, A-8047 Graz, Austria

**Keywords:** Crimson Crisp apples, on-tree maturation and ripening, volatilome, sensory analysis

## Abstract

When new apple cultivars are planted, knowledge of their maturation and ripening behavior and, as a consequence, the best time for their harvest is of utmost importance for providing fruits of the highest quality to consumers. In this study, we followed the on-tree maturation and ripening behavior of organic Crimson Crisp apples over a period of almost 3 months. With the weekly analyses of basic fruit quality attributes (weight, fruit firmness, total soluble solids, titratable acids, starch degradation) in combination with the fruit volatilome (primary and secondary volatiles) and sensory analysis, we obtained a holistic picture of the maturation and ripening properties of this new variety. We could show that at the recommended harvest window, which is based on the degree of starch degradation alone, the fruit development is not finished. Synthesis of aroma volatiles—which is strongly related to the expression of pronounced fruity, apple-like flavor—requires two additional weeks of on-tree ripening. Results indicate an upregulation of 13-LOX at very early maturation stages, while upregulation of 9-LOX and enzyme systems involved in the β-oxidation pathway requires a prolonged on-tree maturation period. The results of this study demonstrate that the analysis of basic fruit quality attributes is not sufficient for understanding the properties of apples. However, we demonstrate that the analysis of the fruit volatilome is a valuable and necessary tool for optimizing the quality of new apple varieties.

## 1. Introduction

Apples (*Malus domestica* Borkh.) are cultivated in almost all parts of the world and they are the most widely cultivated fruit in Europe. They are either marketed as dessert fruits or further processed into a plethora of food products. Many apple cultivars have already been studied intensively and their cultivation together with their fruits/fruit products have been optimized with regard to yield, processability and storability. The overall quality of apple fruits is determined by many different attributes. Appearance, firmness and flavor play a key role in customer acceptance, especially for new apple varieties [1]. The taste of an apple is mostly determined by the levels of soluble sugars and organic acids accompanied by a subtle astringency caused by polyphenols. On the other hand, the apple aroma is composed of odor-active volatile organic compounds [2]. While several hundreds of aroma compounds, including aldehydes, alcohols and esters, have been identified in different apple cultivars, only 30–50 of these compounds are considered to contribute significantly to the distinct aroma of apple fruit [3,4,5].

Many factors influence both the fruit flavor as well as the overall fruit quality. The fruit genetics are the predominant factor for the biochemical flavor formation processes. Therefore, the apple cultivar plays the most important role in the formation of the cultivar-specific properties and the overall fruit quality [6]. However, fruit maturity and ripeness in particular also play a crucial role in flavor formation. Furthermore, external pre-harvest factors such as climate, precipitation and irrigation, sunlight exposure, the composition of the soil but also the application of fertilizers and plant protection agents all affect the apple flavor formation [7].

While abiotic factors and their impact on fruit development can scarcely be regarded as influences, the decision on the harvest time is by contrast an important influence and furthermore one that is chosen by the farmers. In the case of climacteric fruits such as apples, the harvest date has a direct impact on the fruit quality as well as on the biochemical behavior during subsequent storage and/or transportation [8]. As the formation of many flavor compounds starts after the onset of the climacteric rise, the flavor of an apple that is harvested before the climacteric rise will differ significantly from a fruit of the same cultivar that is harvested after the onset of the climacteric rise, or even at the climacteric peak. As the triggered reactions not only impact flavor formation but also many other enzymatically catalyzed reactions such as softening of the fruit tissue, the selection of the harvested date is important for the texture of the fruits. Hence, the selection of an appropriate harvested time is key to obtaining high-quality products [9]. The selection of optimum harvest windows usually requires years of experience with a specific crop. Many tools to establish the optimum harvest window make use of quality attributes such as fruit firmness, sugar content, acidity and starch degradation [10]. To the best of our knowledge, the fruit volatilome has rarely been considered when defining the harvest window for a given apple variety.

The Crimson Crisp, also known as Co-op 39, bred in the United States, is a red-skinned apple variety with a crisp flesh and resulted from a crossbreeding between Golden Delicious and Co-op 17 [11]. The Crimson Crisp apple cultivar has recently found its way into Austrian apple orchards [12]. The antioxidant capacity is reported to be high in comparison to other apple cultivars [13]. In a US study, consumer acceptance for this cultivar was high compared to the traditional and popular variety Jonagold [14]. Crimson Crisp apples are scab-resistant (*V_f_*-gene), making this cultivar ideal for organic cultivation; phylogenetic information of this interesting apple cultivar was described in [13]. This is of particular importance for Austrian apple breeders as around a quarter of all Austrian apples are organically cultivated. At the present time, it is generally recommended that Crimson Crisp apples should be harvested from the early- to mid-season [12]. To the best of our knowledge, these recommendations are mainly based on starch values [15]. In climacteric fruits, such as apples, the onset of enzyme systems relevant to the formation of flavor is specific for each single cultivar [10]. Yet, no data are available for Crimson Crisp apples. The development of volatiles was investigated for Crimson Crisp apples cultivated at different altitudes [15], but during on-tree maturation and ripening. Close observation of volatile and potentially odor-active compound formation during apple development would allow conclusions to be drawn on the onset of enzyme activities.

It was the aim of this study to obtain a deep understanding of the properties and the on-tree ripening behavior of organically grown Crimson Crisp apples cultivated in the south of Austria. To follow the fruit development at different stages of on-tree maturation and ripening, and to determine the optimum harvest window, we investigated Crimson Crisp apples over the time course of a whole vegetation period. Based on preliminary results with Crimson Crisp apples, we hypothesized that monitoring the starch degradation in combination with texture and acidity would not deliver sufficient information to fully describe the on-tree maturation and ripening behavior. Thus, using a holistic approach, we followed the development of the basic fruit quality attributes, the fruit volatilome and also the sensory properties over a period of three months. In addition to the practical application of the obtained results, we aim to demonstrate the importance of flavor analysis and sensory evaluation when new cultivars find their way into commercial orchards.

## 2. Materials and Methods

In this study, we investigated the on-tree maturation and ripening behavior of the scab-resistant apple variety Crimson Crisp. A holistic picture of this cultivar is generated by comparing fruit quality attributes with organic volatile compounds and a sensory evaluation of the fruits. The chosen quality attributes, which help elucidate taste and texture, were weight, starch degradation, total soluble solids, fruit firmness, pH value of the juice and titratable acidity. The volatile organic compounds were studied to evaluate the apple aroma. Sample collection started with harvesting very unripe apples at weekly to 14-day intervals until the fruit was overripe.

### 2.1. Sample Material

Crimson Crisp organic apples grafted on M9 rootstocks were obtained from orchards planted in 2017 at the Research Centre for Fruit Growing & Viticulture, Graz-Haidegg, Austria. The apple cultivation was performed following the guidelines for organic farming (AT-BIO-401 according to EN ISO/IEC 17065:2012).

The recommended harvest date for Crimson Crisp apples [16] was set to be in mid-September. The sampling period was set to 6 weeks before and 6 weeks after this recommended harvest date. To investigate apple development, sampling started on 5 August 2020, 104 days after full blossom (dafb) and ended on 27 October 2020 (188 dafb). Approx. 40 intact Crimson Crisp apples were hand-harvested at weekly to 14-day intervals (i.e., 11 harvests in total). After the harvest, all fruits were immediately brought to the lab and stored at 18 ± 2 °C overnight until further processing.

### 2.2. Analysis of Basic Fruit Quality Attributes

#### 2.2.1. Average Weight

The average fruit weight of the apples was determined from 15–20 intact and undamaged fruits for each harvest time with an analytical scale (PCB, Kern, Balingen-Frommern, Germany, max. 4000 g, d = 0.1 g) and the total weight was divided by the exact number of apples.

#### 2.2.2. Total Soluble Solids Content (TSS)

The total soluble solid content (TSS) was analyzed with a digital refractometer (HI 96814, HANNA Instruments Austria GmbH, Graz, Austria). The refractometer was calibrated before each use with deionized water and triplicates were measured from 2–3 drops of freshly produced fruit juice (see Section 2.3.2). The total soluble solid content is given in °Brix.

#### 2.2.3. Titratable Acidity

Titratable acidity was performed with a commercially available auto-titration system (785 DMP Titrino, Metrohm Inula GmbH, Vienna, Austria). A total of 20 mL of the freshly produced apple juice (see Section 2.3.2) was titrated against 0.1 M NaOH (Convol NORMADOSE, VWR Chemicals, Vienna, Austria) until pH = 10 was reached. The pH value was measured at the beginning of each measurement with the integrated pH electrode and the equivalent point of the titration was calculated in terms of titration volume (mL 0.1 M NaOH). Titratable acidity was calculated from the titration volume and is given in terms of equivalents to malic acid.

#### 2.2.4. Apple Pulp Firmness

After harvest, the pulp firmness of 15 fruits was analyzed using a Pimprinelle II (Art. 27050, Umweltanalytische Produkte GmbH, Ibbenbüren, Germany), which is a fully automated system. The firmness (kg/cm^2^) was measured on two opposing sides of each fruit with an electronic penetrometer with a 1 cm^2^ stamp.

#### 2.2.5. Starch Degradation

To follow the degradation of starch during maturation and ripening, five apples per harvest were cut into halves orthogonally to the apple core. The freshly cut site was dried with a paper towel and dipped into an iodine/potassium iodate solution (Lugol’s solution, 3.3 g/L iodine and 6.7 g/L potassium iodide; Carl Roth, Karlsruhe, Germany) and allowed to rest for 5 min. The color intensity of the blue starch-iodine complex represents the degree of ripening in terms of the apple starch degradation. The coloration pattern was matched with the corresponding degradation grade on a 1–10 point scale (EUROFRU, Ctifl—Centre Technique Interprofessionel des Fruits et Legumes, Paris, France) [17], where grade 1 corresponds to no starch degradation and grade 10 to complete starch degradation in the apple fruits. The mean and standard deviation were calculated from 5 apple halves for each harvest date.

#### 2.2.6. Calculation of the Streif Index

The Streif index (also known as the ripening index or *F*/*RS* value) is a calculated value obtained by dividing the firmness (F) by the product of the TSS value (refractometric value R) and the starch degradation (S) according to Equation (1) and is a dimensionless quantity by definition:

(1) $$\frac{F}{RS}=\frac{Firmness}{Total\;soluble\;solids\cdot Starch\;degradation}$$

### 2.3. Analysis of the Fruit Volatiles

#### 2.3.1. Sample Preparation for the Analysis of Primary Aroma Compounds

For the analysis of primary aroma compounds, the inactivation of apple native enzymes must be achieved as quickly as possible to prevent the formation of secondary aroma compounds. To achieve this, we followed a protocol described by Aprea et al. [18].

Following this protocol and to obtain a representative sample, 8 to 10 fruits were peeled and cut into pieces. Immediately after cutting and peeling, an aliquot of approx. 75 g flesh was inserted into a solution prepared from 75 mL deionized water, 30 g NaCl (≥99.5%, Merck, Darmstadt, Germany), 250 mg citric acid monohydrate (puriss., Sigma-Aldrich, Germany) and 250 mg L-ascorbic acid (≥99.8%, Sigma-Aldrich, Germany) to inactivate the enzymes. The mixture was homogenized in a commercial blender (ProBlend 6, Philips, Vienna, Austria) to purée-like samples. Aliquots of this mixture were immediately deep frozen in glass vials and stored at −25 °C until further use.

#### 2.3.2. Sample Preparation for the Analysis of Secondary Aroma Compounds

Apple juice was prepared from approx. 500 g freshly cut and peeled apple pieces from 8–10 fruits with a commercial fruit juicer (HR1921/20; Philips, Vienna, Austria). After, the samples were left to rest for 30 min in closed glass vials and aliquots were deep frozen at −25 °C until further use.

#### 2.3.3. Analysis of the Apple Volatiles by HS-SPME-GC-MS

For the analysis of primary aroma compounds, aliquots of the homogenized samples (250 mg purée) were transferred into 20 mL headspace vials. 2-Octanol (purity > 99%, Sigma Aldrich, Taufkirchen, Germany) was used as an internal standard (30 ng absolute in methanol, purity > 99%, Fisher Scientific, Schwerte, Germany). For the analysis of secondary aroma compounds, 100 µL of apple juice was used with the addition of 100 mg NaCl and 2-octanol as an internal standard (30 ng absolute in methanol) in 20 mL headspace vials. All samples were analyzed in duplicate in randomized order. The extraction/enrichment of the volatiles was performed by headspace solid-phase micro extraction (HS-SPME) using a CTC Combi PAL sampler (CTC Analytics, Zwingen, Switzerland). A 50/30 µm DVB/Car/PDMS 2 cm stable flex SPME fiber (Supelco, Bellefonte, PA, USA) was used for the enrichment of the volatiles. Prior to the extraction of the volatiles, the samples were equilibrated in the oven of the autosampler at 40 °C for 5 min and the fiber was exposed in the headspace of the equilibrated sample at 40 °C for 20 min. Samples were stirred thoroughly during the equilibration and enrichment process. Immediately after the exposure, the fiber was transferred into the injector of the GC system for thermo-desorption. The SPME fiber was left in the injection port for re-conditioning (20 min) before it was exposed to the headspace of the next sample. Analysis of the volatiles was performed with one-dimensional gas chromatography-mass spectrometry (GC-MS) (Shimadzu GCMS-QP 2010 Plus, Shimadzu Europa GmbH, Duisburg, Germany) on a semi-polar column (Rxi^®^ 5 ms, 30 m × 0.25 mm × 1 µm, Restek Corporation, Bellefonte, PA, USA). The following conditions were used: a temperature program starting at −10 °C for 1 min with a temperature ramp of 8 °C/min up to 270 °C (1 min holding time). Cryo-focusing was performed to achieve an improved separation for highly volatile compounds. The start temperature of −10 °C was reached by blowing liquid nitrogen into the GC oven using a specific cryo vent attached to the GC oven. Splitless injection with a sampling time of 1 min and an injection temperature of 270 °C was performed using an SPME liner with a constant inner diameter of 0.75 mm in the GC injection system. Helium was used as a carrier gas with a linear velocity of 35.0 cm/s. Mass-selective detection was performed in the scan mode (35–350 *m*/*z*, EI (70 eV), interface temperature 280 °C, ion source temperature 200 °C).

The obtained chromatograms were analyzed with the deconvolution software PARADISe [19], which is based on the PARAFAC2 model [20]. The identification of the compounds was performed on probability-based matching of the mass spectra to those from MS libraries (NIST14, Adams Essential Oils library, FFNSC 4.0) or authentic reference compounds as well as on linear-temperature programmed retention indices (RI) [21]. RI were calculated using the homologous series of n-alkanes (C5-C26) and were compared to RI from authentic reference compounds or data from the literature. The relative concentrations (semi-quantification) were calculated by comparison of the respective peak areas obtained from the total ion chromatograms after deconvolution to that of the internal standard 2-octanol assuming a response factor of one for all compounds according to Elmore (2015) [22]. Relative concentrations of primary aroma compounds were calculated from the sample amount considering the relative fruit content of the purée and are given in mg/kg. Relative concentrations of secondary aroma compounds were calculated directly from the juice and are given in mg/L juice. Cumulative concentrations are calculated by summing up all relative concentrations of one compound group for one harvest. The relative contribution of different volatile fractions is calculated by dividing the sum of all relative concentrations of one compound group by the sum of all relative concentrations of all compounds for each harvest.

### 2.4. Sensory Analysis

#### 2.4.1. Panel Training and Selection

Sensory analysis was performed by a well-trained panel under standardized conditions in the sensory laboratory. All panelists (five females and two males aged between 25 and 51 years) fulfilled the basic requirements given by DIN EN ISO 8586 and all but two had several years of experience conducting sensory evaluation (between 5 and 20 years). Additionally, training sessions specific to apple flavor were carried out prior to the study. This training sensitizes the panelists to the expected sensory impressions during apple analysis. On the one hand, the training focused on volatiles as well as taste stimuli present in apples; on the other hand, the panelists were trained in using the check-all-that-apply method (CATA) (see Section 2.4.3). For the aroma training, solutions of relevant compounds were prepared in ethanol in adequate concentrations (1% unless indicated). Filter strips were dipped into these solutions and dried and stored in cellophane coats until sensory evaluation. The following compounds were used for the odor training: ethyl decanoate, α-ionone (2%), α-terpineol, linalool, 5-hexenyl acetate, hexyl butanoate, 2-hexenal (*E*), 3-hexenol (*Z*), ethyl butanoate and β-damascenone. At the time of the investigation, all compounds were registered in the European Union as flavoring compounds and authorized to be used in flavored foods according to regulation (EU) No. 872/2012. The compounds were purchased from Sigma-Aldrich (Vienna, Austria) and had a purity of ≥ 96% (food-grade quality). Apple juice samples with adjusted sweetness and acidity were prepared for the taste evaluation training. The panelists were asked to rank four apple juice samples according to their increasing sensation of sweetness (sucrose addition: 1.: +1 g/L, 2.: +5 g/L, 3.: +10 g/L, 4.: +15 g/L) and acidity (citric acid addition: 1.: +0.1 g/L, 2.: +0.5 g/L, 3.: +1.0 g/L, 4.: +1.5 g/L), respectively. Furthermore, apples of different varieties, origin and degrees of ripeness were presented to the panelists in two sessions for sensory evaluation to train on the CATA method. The results of the versatile evaluations were discussed among the panel members. With respect to CATA, the discussion on relevant descriptors resulted in a list of 28 attributes covering a broad range of apple characteristics. The final selection of the panelists was made according to their ability to describe the apple samples.

#### 2.4.2. Sample Preparation for Sensory Evaluation

Intact apples were washed and cut into 12 pieces each. The stem, core and seeds were removed and the apple slices were dipped into an antioxidant solution (0.2% citric acid, 0.2% ascorbic acid, 0.5% calcium chloride; 98%, Merck, Darmstadt, Germany) for 30 s to avoid oxidation/browning of the surface [23]. A minimum of three apple slices of three different apples was offered to each panelist in a sealed cup with a randomized 3-digit code for each sample. Samples were served in a randomized order.

#### 2.4.3. Sensory Evaluation of Apples to Follow On-Tree Maturation/Ripening via CATA

Check-all-that-apply (CATA) was chosen for the evaluation of the apple samples from different harvest dates. The panel evaluated the apples from all harvest dates between 139 and 174 dafb. CATA allowed us to quickly assess a broad range of sensory attributes. While this technique is primarily used with consumers, examples from oenology and also from our own previous research have proved to give good results when CATA is combined with a lower number of trained panelists [24,25,26].

Sensory evaluation was carried out after each harvest from 139 to 174 dafb resulting in 5 sessions. Due to a very limited number of apple samples, sensory evaluation was not possible at the end of the whole experiment (i.e., 188 dafb). The following 28 sensory attributes were selected for the evaluation of the on-tree maturation experiment: firm, juicy, sweet, bitter, fresh apple juice, crisp, soft, odorless, tasteless, mealy, apple odor, astringent, citrus notes, floral notes, very sweet, green/grassy, sour, very sour, ripe, unripe, overripe, honey, fresh, banana, balanced sweet-acid, unbalanced sweet-sour, stale and fruity. Data collection was performed with the use of Compusense software (Compusense, Guelph, ON, Canada).

### 2.5. Statistical Data Treatment

The statistical analysis of the data was performed with the MS Excel add-in XLSTAT (Addinsoft (2021), XLSTAT statistical and data analysis solution. Long Island, NY, USA). For the identification of significant concentration differences between the samples and their maturation/ripening stages, one-way analysis of variance (ANOVA) was performed (*p* = 0.05). If statistically significant differences were observed within the data sets, the ANOVA was followed by post hoc pairwise comparison using Tukey’s honestly significant difference (HSD) test control (*p* < 0.05) to check for differences between single samples. Correlations between samples and volatiles were analyzed with a principal component analysis (PCA) using the Pearson correlation. For the evaluation of results obtained from CATA experiments, correspondence analysis with Cochran’s Q test (*p* = 0.05) was applied with subsequent multiple pairwise comparison applying the McNemar test with Bonferroni correction. Principal coordinates analysis (PCoA) was applied to determine the correlation coefficients for visualization in a two-dimensional map.

## 3. Results and Discussion

To gain a holistic view of the on-tree maturation of the organically grown scab-resistant apple variety Crimson Crisp, a wide spectrum of quality attributes was investigated for each harvest time over a period of 13 weeks. For a deep understanding of the maturation and ripening behavior of this apple variety, we investigated basic quality attributes such as fruit weight, starch gradation and firmness and the correlation with volatile profiles and sensory quality.

### 3.1. Fruit Quality during On-Tree Maturation

Fruit quality attributes such as fruit weight, total soluble solids, pH-value of apple juice, titratable acidity, fruit firmness, starch content and Streif index of apples harvested from 104 dafb (green fruit) to 188 dafb (overripe) are given in Table 1.

During on-tree maturation and ripening of Crimson Crisp apples, a steady increase was observed for the average fruit weight from 128.2 g at the beginning to 223.3 g at 160 dafb. After this point, the fruit development was completed and the weight stayed constant between 222 and 236 g until the end of our harvest period. The starch degradation developed from a score of 1.6 at 104 dafb, which is characteristic of unripe fruits [27], followed by starch degradation until reaching a score of 10.0 at 188 dafb. The highest increase occurred between 132 and 146 dafb, from 3.4 to 7.2, respectively, within 2 weeks only. According to current harvest recommendations, the apples were ready to be picked at that period [11,12].

A steady rise of the TSS values was observed until 153 dafb. After this date, the TSS content stayed almost constant with a minor increase of approximately 0.4 °Brix during the last three weeks, remaining constant at 14.1 °Brix until the end of the harvest period. These observations are in accordance with previously published data for other apple cultivars [28,29]. Since sugars comprise the major portion of soluble solids in apples, the measurement of TSS is an excellent tool for estimating the sweet sensation when consuming apples or apple juice [30]. With respect to fruit firmness, small changes between harvests were observed with an overall decrease in firmness during maturation and ripening. Fruit firmness gives information about the texture and crispness of apple fruit. However, based on the texture values alone, crisp apples cannot be differentiated from mealy fruits. This differentiation requires sensory evaluation [31].

Titratable acidity of fruit juice is typically used to assess the perceived tartness of apple [30]. Beginning with 118 dafb, we observed a steady decrease in TA, which is an indicator of fruit maturation. The highest decline in TA (26%) was observed between 139 and 146 dafb. The observed decrease in TA is accompanied by the corresponding increase in pH value. Organic acids are converted into sugars at later stages of apple maturation, which leads to a decline in TA concentrations and a parallel increase in the pH [32,33].

However, to the best of our knowledge, no general F/RS value recommendations for Crimson Crisp apples have yet been reported. Recommended F/RS values can be found in the literature for a large number of apple varieties as guidelines for determining optimum harvesting times [34]. In general, common recommendations for harvest windows range from F/RS = 0.3 at the upper end to F/RS = 0.06 at the lower end [34]. It is important to note that the recommended F/RS are cultivar specific and strongly depend on whether the apples are to be stored or are intended for direct consumption or processing [34]. In general, F/RS values decrease steadily with increasing ripeness and maturity, respectively, and can also be used to predict the quality of fruits [34]. This behavior was also observed in this study with an F/RS value of approximately 0.7 at 104 dafb and a final value of 0.06 at 188 dafb.

However, these values alone do not provide sufficient information to judge the development of this cultivar but require a thorough investigation of the fruit volatiles and sensory properties.

### 3.2. Development of Volatile Compounds during On-Tree Maturation

Several biosynthetic pathways are known for the formation of volatile and potential aroma compounds in fruits. For apples, the degradation of fatty acids via the β oxidation and lipoxygenase (LOX) pathways as well as the amino acid degradation pathway during later stages of fruit ripening play the predominant roles, while compounds that are derived from carbohydrates as precursors are of minor importance in apple [10]. The expression of sensory properties in different apple cultivars is genetically controlled and thus, different apple cultivars may show different ripening behaviors [6]. The production of the volatiles is a function of the activities of the respective enzyme systems and the precursor availability. Several studies have previously reported variety-specific onsets on maturation/ripening patterns, and thus differences in the up- and down-regulation of the enzymatic systems that are relevant for the production of flavor compounds [10]. As the synthesis pathways for apple flavor compounds are well understood, we did not analyze the enzyme systems in this study, but we focused on the volatiles, which are distinctive to the specific behavior of Crimson Crisp apples.

**Figure 1:**
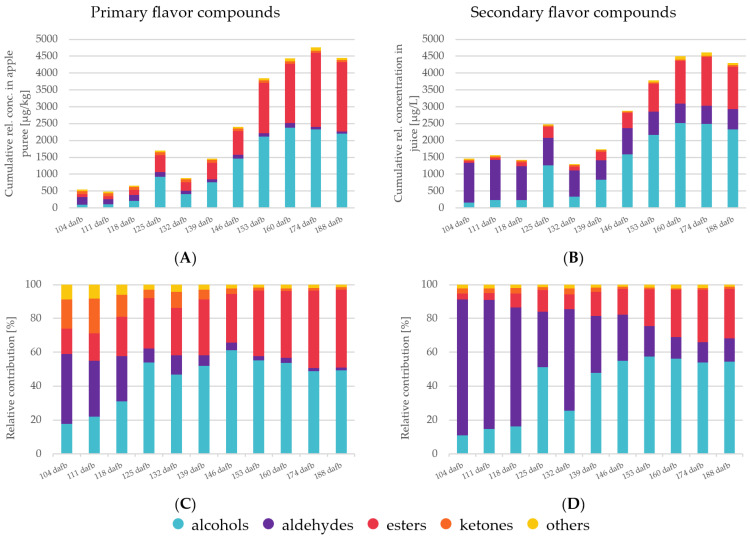
The cumulative concentration and relative contributions of different compound classes is depicted in four diagrams. (**A**)—relative cumulative concentration of primary volatiles; (**B**)—relative cumulative concentration of secondary volatiles; (**C**)—relative contribution of the compound classes to the rel. cumulative concentration in primary volatiles; (**D**)—relative contribution of the compound classes to the rel. cumulative concentration in secondary volatiles.

The analysis of the volatile fraction of apple fruits from each harvest point serves to elucidate the aroma profile of Crimson Crisp apples. In this study, primary aroma compounds were investigated after immediate inactivation of the enzymes that are genuine native properties of the fruit; secondary aroma compounds were analyzed from freshly produced apple juice, respectively. In total, 79 primary aroma compounds (i.e., 15 alcohols, 13 aldehydes, 31 esters, 9 ketones and 12 compounds from other classes) (Table 2) and 77 secondary aroma compounds (i.e., 23 alcohols, 11 aldehydes, 29 esters, 7 ketones and 6 compounds from other classes) (Table 3) were identified. A detailed list of all volatile compounds, their linear temperature-programmed retention indices and their mean relative concentrations can be found in Table 2 and Table 3. Figure 1 shows a summary of compound classes and their relative changes in the course of the on-tree fruit development, their cumulative concentrations and their relative contribution to the primary and secondary volatilome. The synthesis of volatiles over time and also the change in distribution among the different compound classes can be seen clearly. That is, a maximum of volatile compounds was observed at very late ripening stages (174 dafb) with an on-set of flavor formation between 130 and 140 dafb. Considering the recommended harvest date of 130–140 dafb [11], it is obvious that apples harvested at this early ripening stage will only contain a low overall concentration of volatile compounds.

### 3.3. Correlation of Volatiles with Quality Attributes via Principal Component Analysis

**Figure 2:**
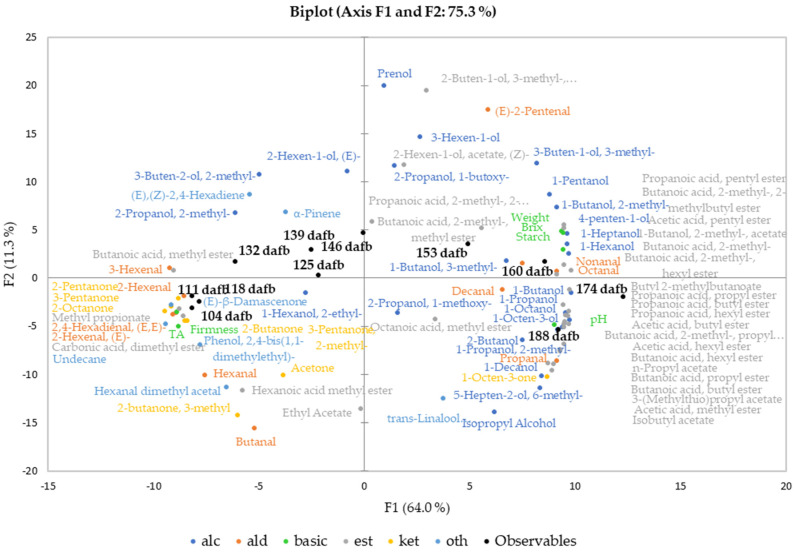
Principal component analysis (PCA, Pearson correlation) biplot of basic fruit quality attributes and the volatilome (2° aroma compounds) of Crimson Crisp apples (variables) and the harvest date (observables). The harvest dates (observables) are black and form a clockwise pattern starting in quadrant III, passing through II and I and ending in IV.

Principal component analysis (PCA) was conducted to investigate the correlation between fruit quality attributes and the volatile compounds at different harvest dates of Crimson Crisp apples. Figure 2 shows the biplot for the first two principal components of the correlation of the basic fruit quality attributes and the secondary aroma compounds over the harvest period. PCA analysis including the primary aroma compounds delivered comparable results and is, thus, not shown.

The biplot shows a clockwise development of sampling points and analyzed quality attributes starting in quadrant III at 104 dafb turning approx. 270 degrees to the end of the harvest period at 188 dafb. The first group of samples harvested between 104 and 118 dafb forms a dense cluster in the third quadrant of the PCA, showing close correlation with each other. The clockwise trend continues in the second quadrant, the observables 125–146 dafb are spread chronologically. However, these show less correlation to one another than the earlier sampling points, suggesting that the fruits collected at these harvest dates and beyond may be differentiated better compared to the earlier harvest dates. These dates in the middle of the harvest period, the timeslot for the current harvest recommendation, represent the final stages of fruit maturation. The harvest dates 153 and 160 dafb are found in quadrant I of the biplot; 174 and 188 dafb are located in quadrant IV (Figure 2).

The values for the basic fruit quality attributes (Table 1) were included in the multivariate data analysis together with the relative concentrations of the volatile compounds. The basic fruit quality attributes such as weight, starch degradation and TSS were closely correlated to the last three harvest dates. Interestingly, the fruit weight, the TSS and also the starch degradation were related closest to 160 dafb, indicating that the physiological development was completed at that point of the vegetation period. The quality attributes TA and fruit firmness are located in the third quadrant as well, since higher firmness and acid content are linked to unripe fruits [35]. However, the constantly increasing pH value in combination with the ongoing development of flavor compounds indicates further ripening processes until the end of our harvest window at 188 dafb.

Aldehydes are volatile compounds produced via the LOX pathway from free fatty acids [10]. The observed differences in relative concentrations between primary and secondary aroma compounds by a factor of approximately 5 are based on the release of LOX on cell disruption during the processing of the juices [18]. In Crimson Crisp apples, the overall relative concentrations of aldehydes were higher at earlier stages of maturity than in later ones (Figure 1). This corresponds well to previously reported results [10,15]. In both cases, primary and secondary aroma compounds, it can be clearly seen that the overall aldehyde concentrations were abundant in the unripe fruits with significantly lower concentrations in absolute values as well as the relative share of the total volatilome in the ripe fruits (Figure 1). C6 and C9 aldehydes are formed from the corresponding fatty acids upon activities of the respective enzyme systems (i.e., LOX and hydroperoxide lyases HPL) [36,37]. In the PCA biplot, high correlations of C6 aldehydes such as 2-hexenal (*E*), 2,4-hexadienal (*E*, *E*), 2-hexenal (*E*) or 3-hexenal (*Z*) were observed, whereas, for example, the C9 aldehyde nonanal was strongly correlated with the sample from late harvest dates. This indicates that the activities of 13-LOX and 9-LOX in combination with HPL are most probably upregulated at different ripening stages, 13-LOX being active already at very early maturation stages followed by a late upregulation of 9-LOX towards the end of the experiment [10]. In the present study, 2-Pentenal (*E*) is located in quadrant I with a closer correlation to higher ripening stages than earlier ones (Figure 2). The formation pathways of C5 LOX products have not been elucidated as yet in previous work [38].

In comparison to aldehydes, alcohols are less pronounced in unripe fruits both in absolute quantities and also in relation to the other compound classes (Figure 1). In the PCA biplot (Figure 2), the majority of volatile alcohols are found in quadrants I and IV and arranged on an almost straight vertical line. This behavior indicates that they are closely correlated to each other in the first dimension, which describes the largest differentiation between unripe and ripe fruits. Nevertheless, further differentiation between many alcohols can be observed along the F2 axis, indicating further development with increasing ripeness. The compound 1-butanol derived from either β-oxidation or LOX with the highest relative concentration (Table 3) is located in quadrant IV, centroid to the three observables 160, 174 and 188 dafb. The compound 1-hexanol, which is synthesized via β-oxidation or LOX [10], and the compound 2-methyl-1-butanol, a degradation product of the amino acid isoleucine [10], are found in quadrant I near 160 dafb. This indicates an upregulation of the involved enzyme systems (i.e., alcohol acyl CoA transferase and the amino acid specific aminotransferase in combination with alcohol dehydrogenase [39]) in later ripening stages. Relative concentrations of approx. 100–200 µg/kg were found in primary aroma compounds during the first three weeks of harvest, taking a share of 18–31% of the total volatile fraction. A similar behavior was found for alcohols as part of the secondary aroma compounds; however, their percentage share did not exceed 16% of the total volatilome from 104 to 118 dafb. During later ripening stages, alcohols became the most abundant group of compounds in the volatilome of Crimson Crisp apples. Starting at 139 dafb, a clear and steady onset of volatile alcohols was observed and from 153 to 188 dafb alcohols were present in relative concentrations of >2000 µg/kg apple pulp, and the percentage of alcohols in the volatilome was between 49–55%. Looking at the secondary aroma compounds, the pattern was similar with a relative concentration of higher than 2000 µg/L alcohols in juices from 153 dafb and later during the experiment. The following studies have investigated the activation of different enzymes in apples over the course of maturation and ripening. Our observations of volatile compounds are in accordance with the reported data on enzyme regulation in Golden Delicious and Jonagold apples, particularly for the activities of the lipoxygenase (LOX) [40] and alcohol dehydrogenase (ADH) [41]. Additionally, the amino transferase activity is also reported to increase during fruit development in Fuji apples, and thus the increase of branched alcohols [42].

Next to alcohols, volatile esters constitute another important compound class as well as the largest cluster in the PCA biplot. In general, volatile esters play an important role in the formation of fruit flavor as most of them show distinct fruity character [43]. Esters are formed via two major pathways, (i) via β-oxidation upon the oxidative breakdown of the fatty acids resulting in C4 and C6 straight chain methyl esters and ethyl esters as well as acetates and (ii) as the final results of the amino acid degradation forming esters of methyl-branched butanoic acid or of propanoic acid. In our study, the overall concentration patterns of esters show a shape that is comparable to those of alcohols. In ripe Crimson Crisp apples, the most abundant esters were butyl acetate, hexyl acetate as well as 2-methylbutyl acetate, which is characteristic of many apple varieties [27]. Esters, with some minor exceptions, such as methyl hexanoate or methyl butanoate as β-oxidation products which are already formed in the early stages of maturity, show a close correlation with apples harvested at 160, 174 and 188 dafb. Their close correlation with the ripe fruits demonstrates the importance of long on-tree ripening for the formation of a pronounced apple flavor. A previous study described a slight decrease in the activity of alcohol acyltransferase (AAT) and pyruvate decarboxylase (PDC) in the pulp during ripening in Golden Reinders apples [35]. However, advanced ripeness of the fruits leads to an increased bioavailability of precursors leading to high ester concentrations and thus to high fruitiness of the ripe products [44]. On the other hand, many enzymes involved in the bio-formation of esters, such as ADH and LOX, demonstrate higher activity in mature fruits compared to unripe Fuji and Ruixue samples [42]. With respect to the current harvest recommendation, the given correlations in Figure 2 show very clearly that for the formation of fruitiness, Crimson Crisp apples require longer on-tree ripening than approx. 135 dafb.

Some ketones were identified as primary and secondary aroma compounds in Crimson Crisp apples. However, they are present in only low concentrations (Figure 1). Their relative share diminishes from approximately 20% (primary volatiles) and 3% (secondary volatiles) at early harvest dates to only approx. 1% of the volatilome in both categories. Ketones are also not known for outstanding sensory properties [45]. Thus, we do not expect high impact of this compound class on the aroma of Crimson Crisp fruits, especially at the low concentrations. Volatiles from other categories, such as (un)saturated hydrocarbons or terpenes also play a minor role in the volatilome of Crimson Crisp apple pulp in terms of their relative concentrations. (E)-β-damascenone with its very low odor threshold value and its distinct apple notes is reported to be a key component in many apple varieties [46]. In this study, (E)-β-damascenone was identified in all samples; however, no clear trend was observed in relation to the maturation or ripening stage.

### 3.4. Sensory Quality

**Figure 3:**
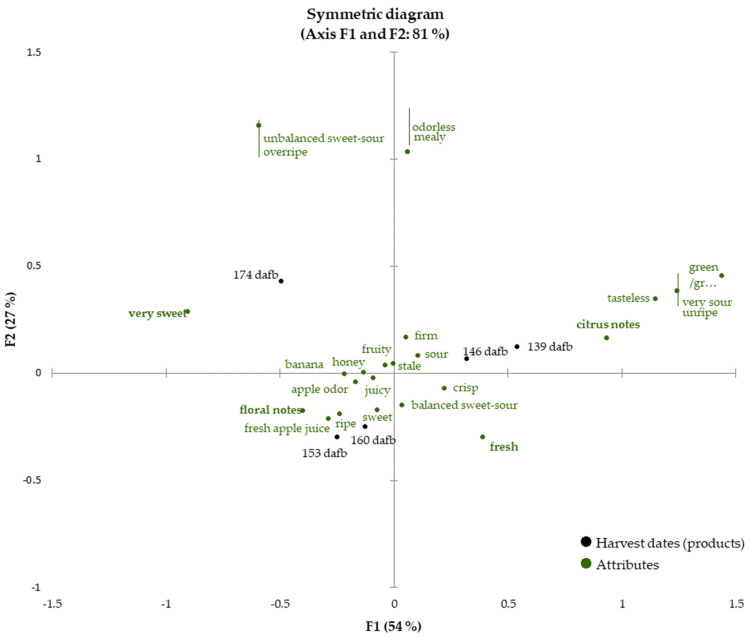
Check-all-that-apply (CATA) analysis of the apples harvested between 139 to 174 dafb. The harvest dates (products) are printed in black, all attributes are colored in green. Discriminating attributes (*p* < 0.05) are printed in bold.

Once the fruits reached eating quality (beginning with 139 dafb), sensory analysis of the Crimson Crisp apples was carried out for each harvest date until 174 dafb to follow the development of the sensory properties and to correlate them with the data presented in the previous sections. The check-all-that-apply (CATA) technique was conducted by evaluating 28 descriptors (see Section 2.4.3. for the detailed list of sensory attributes). The sensory attributes cover a broad range of properties describing odor, texture and taste to obtain a holistic picture of the sensory properties. Figure 3 shows a biplot as the result of CATA analysis for the development of the sensory properties during apple on-tree ripening from 139 dafb to 174 dafb (81% of explained total inertia on the first two dimensions with F1 54%, F2 27%). The sensory attributes soft, bitter and astringent were not selected by any panelist and consequently are not presented in the CATA chart. For better visibility, attributes showing the same eigenvalues are marked with red lines to indicate that two different sensory attributes correspond to one point in the biplot.

The two earliest harvest dates, which correspond to current harvest recommendations, 139 and 146 dafb are located in quadrant I closely related to the sensory attributes ‘(very) sour’, ’tasteless’, ‘unripe’ and ‘firm’, indicating low ripeness of the apples, which demonstrates that the fruits are not sufficiently ripe for harvest and that the current harvest recommendation is set as too early. The attributes ‘firm’, ‘sour’, ‘fresh’ and ‘a balanced sweet-sour ratio’ are located in between the later harvest dates 146 and 160 dafb. Within this period, the taste of the fruits changed from ‘very sour’ to ‘sour’, indicating a ripening process of the fruits. It is interesting to note that very few odor descriptors correlate with the earlier harvest dates, but are dominated by sensory attributes describing taste and texture. Fruits from this ripening stage only exhibit approx. 50% of the total volatiles compared to later ripening stages (Figure 1). The predominance of ‘green/grassy’ and ‘citrus’ can be well explained by the high quantities of C6 aldehydes in the early ripening stages.

In chronological order, the next group of harvest dates is located in quadrant III. The apples harvested at 153 dafb and 160 dafb were very similar, and thus closely correlated to each other in the biplot. Sensory attributes, which can be associated with flavorful and ripe apples, are correlated to a great extent with sampling dates 153 and 160 dafb. Smell and taste attributes such as ‘apple odor’, ‘fresh apple juice’, ‘sweet’ and ‘fruity’ can be found in this area of the biplot as well as the attributes ‘juicy’ and ‘ripe’. The high correlation with the descriptors ‘honey’, ‘banana’ and ‘floral notes’ highlight the intense aroma of ripe Crimson Crisp apples. The similarities in the sensory properties are also reflected in the results obtained from volatilome analysis as the overall volatiles quantities are comparable in fruits harvested at 153 and 160 dafb, respectively. The latest harvest date under investigation (i.e., 174 dafb) is located in quadrant II. The attributes ‘very sweet’, ‘unbalanced sweet-sour impression’ and ‘overripe’ are found in quadrant II as well, even though the correlation with 174 dafb is lower than for earlier harvest dates and sensory attributes. Although the overall content of volatiles was high, the CATA results suggest, that this harvest date is less related to odor-associated descriptors than to taste and texture sensory attributes that indicate very high ripeness and the onset of senescence.

The prolonged on-tree maturation improves the flavor properties of this apple cultivar. As given in its name, crispiness is a characteristic of this apple cultivar and is maintained for a long ripening period.

## 4. Conclusions

In this study, we performed a holistic investigation of the on-tree maturation and on-tree ripening behavior of the scab-resistant apple variety Crimson Crisp. Harvest recommendations for farmers are frequently given on the basis of experiences with other apple cultivars grown under the same conditions and are mostly based on analytical quality attributes that can be easily determined. Results from this study showed that it is crucial to perform thorough investigations of the fruit properties when new cultivars are introduced to apple orchards.

From the observation of the quality attributes we can conclude that the biggest changes in terms of fruit maturation and ripening processes occur between 132 to 146 dafb followed by a significant reduction in reaction rates at later harvest times. When relating the time scale of the fruit development with the recommended harvest date of between 130 and 140 dafb, it is clearly apparent that the fruit has developed well until this date. However, longer on-tree maturation does not lead to rapid degradation of the structure or even senescence of the fruit. The analysis of the fruit volatilome reveals that the synthesis of many aroma compounds can be increased if the apples are let to ripen longer on-tree. The formation of esters, and thus the fruity character, requires an on-tree period of at least 160 dafb. The evaluation of the sensory quality shows a similar trend. Many favorable sensory attributes show their highest correlation to the harvest at 153 and 160 dafb, while earlier and also later harvests lack a close correlation to descriptors associated with desired flavor descriptors.

For Crimson Crisp apples, the results show that the current harvest recommendations given in our geographical region should be prolonged to approximately 155–160 dafb to significantly increase the perceived fruit quality. To complete the evaluation of the scab-resistant cultivar Crimson Crisp, a study should be conducted to evaluate the storage properties, focusing on the later harvest dates.

Using Crimson Crisp apples, we were able to show that in order to find optimum harvest windows for a given apple variety, it is not sufficient to follow the starch indicator alone. The use of flavor analyses and sensory evaluation delivers valuable information on the cultivar of interest. Furthermore, we demonstrate that sensory analysis must be an indispensable part of fruit evaluation. Analysis of the fruit volatiles indicates high concentrations of value-determining compounds such as fruit esters; however, the onset of senescence could only be determined by the combination with sensory evaluation delivering the overall sensory impression of the fruit.

Apple breeders work continuously on the breeding of new apple varieties with improved properties that fulfill consumer demands and that are suitable for cultivation under present-day conditions in orchards. Our study can also be seen as an appeal to fruit growers and also to people from fruit trading or fruit processing companies not to rely simply on copying their experiences from traditionally cultivated varieties, but rather to involve themselves intensively with new cultivars in order to take full advantage of the valuable properties these can provide.

## Figures and Tables

**Table 1:** Quality attributes of Crimson Crisp apples harvested between 104 to 188 dafb.

Dafb	Weight [g]	Starch [1,2,3,4,5,6,7,8,9,10]	TSS ^a^ [°Brix]	Firmness [kg/cm^2^]	pH ^b^	TA ^c^ [g/L Malic Acid Equiv.]	F/RS ^d^
**104**	128.2	1.6	10.0	11.4	3.11	10.6	0.72
**111**	125.1	2.0	10.0	10.6	3.06	8.47	0.53
**118**	135.9	2.0	10.9	10.7	3.11	10.1	0.49
**125**	154.6	2.8	11.6	10.3	3.17	8.93	0.32
**132**	178.3	3.4	11.1	9.58	3.16	8.26	0.25
**139**	181.2	6.0	12.5	8.81	3.18	8.11	0.12
**146**	193.6	7.2	12.8	9.24	3.32	6.03	0.10
**153**	205.5	7.8	13.7	8.51	3.30	5.91	0.08
**160**	223.3	8.4	13.7	8.68	3.32	5.59	0.08
**174**	236.4	9.8	14.1	8.01	3.53	5.56	0.06
**188**	222.2	10.0	14.1	8.02	3.64	4.39	0.06

^a^ TSS = total soluble solids, ^b^ pH = pH-value, ^c^ TA = titratable acid, ^d^ F/RS = Streif index.

**Table 2:** Relative concentrations (average values, *n* = 2, µg/kg) of the volatile compounds (1° aroma compounds) identified in fruit purée of Crimson Crisp apples after enzyme inactivation harvested at different stages of maturity/ripeness (104–188 dafb).

Compound Name ^a^	RI [DB5] (exp.) ^b^	RI [DB5] (lit.) ^c^	104 dafb ^d^ [µg/kg]	111 dafb ^d^ [µg/kg]	118 dafb ^d^ [µg/kg]	125 dafb ^d^ [µg/kg]	132 dafb ^d^ [µg/kg]	139 dafb ^d^ [µg/kg]	146 dafb ^d^ [µg/kg]	153 dafb ^d^ [µg/kg]	160 dafb ^d^ [µg/kg]	174 dafb ^d^ [µg/kg]	188 dafb ^d^ [µg/kg]
***Alcohols***													
2-Propanol	510	506	0.80	0.93	1.26	1.17	0.87	1.21	0.99	1.18	1.06	1.03	1.41
1-Propanol	568	558	0.10 ^F^	0.08 ^F^	0.11 ^F^	9.8 ^D^	1.29 ^F^	4.12 ^E^	10.3 ^D^	22.8 ^C^	20.4 ^C^	30.6 ^A^	26.3 ^B^
2-Butanol	607	609	0.45 ^G^	0.58 ^FG^	0.65 ^EFG^	1.01 ^B^	0.70 ^EF^	0.75 ^CDEF^	0.95 ^BC^	0.73 ^DEF^	0.84 ^BCDE^	0.93 ^BCD^	1.43 ^A^
3-Buten-2-ol, 2-methyl-	617	614	2.41 ^CD^	3.24 ^AB^	3.18 ^AB^	2.79 ^ABCD^	2.64 ^BCD^	2.85 ^ABC^	3.39 ^A^	2.54 ^BCD^	2.30 ^CD^	2.06 ^D^	2.16 ^CD^
1-Propanol, 2-methyl	634	635	1.83 ^I^	2.06 ^I^	2.76 ^HI^	12.3 ^F^	4.70 ^H^	8.51 ^G^	16.6 ^E^	22.1 ^D^	25.4 ^C^	32.4 ^B^	41.4 ^A^
1-Butanol	670	670	6.83 ^I^	11.0 ^I^	22.6 ^HI^	250 ^F^	56.6 ^H^	159 ^G^	331 ^E^	580 ^D^	699 ^C^	812 ^A^	774 ^B^
1-Butanol, 3-methyl-	738	738	2.05 ^DE^	2.75 ^C^	1.66 ^E^	3.79 ^A^	1.72 ^E^	1.83 ^E^	3.37 ^AB^	2.95 ^BC^	2.84 ^BC^	2.57 ^CD^	2.54 ^CD^
1-Butanol, 2-methyl-	743	740	22.7 ^H^	31.9 ^H^	72.8 ^G^	373 ^D^	232 ^F^	324 ^E^	619 ^C^	757 ^A^	788 ^A^	695 ^B^	653 ^C^
1-Pentanol	769	771	11.6 ^H^	10.7 ^H^	11.7 ^H^	29.8 ^F^	18.2 ^G^	38.8 ^E^	74.9 ^D^	99.5 ^B^	110 ^A^	80.6 ^C^	75.5 ^CD^
2-But-2-en-1-ol, 3-methyl	777	772	2.52 ^H^	3.37 ^GH^	4.34 ^FG^	10.3 ^D^	11.3 ^D^	18.4 ^B^	38.5 ^A^	15.0 ^C^	10.1 ^D^	7.26 ^E^	5.61 ^F^
2-Hexen-1-ol, (*E*)-	868	867	20.9 ^F^	16.2 ^GH^	39.1 ^B^	36.3 ^C^	23.7 ^DE^	22.2 ^EF^	42.6 ^A^	25.7 ^DE^	21.3 ^F^	18.2 ^G^	15.4 ^H^
1-Hexanol	870	870	16.5 ^G^	15.7 ^G^	38.8 ^FG^	184 ^E^	57.2 ^FG^	173 ^E^	321 ^D^	583 ^C^	687 ^A^	632 ^B^	592 ^C^
2-Propanol, 1-butoxy-	945	947	1.33 ^AB^	1.18 ^AB^	1.46 ^AB^	1.53 ^AB^	0.95 ^B^	1.30 ^AB^	1.21 ^AB^	1.53 ^AB^	1.73 ^A^	1.90 ^A^	1.90 ^A^
Pentan-1-ol, 2-ethyl-4-methyl ^t^	956	n.a.	1.15 ^A^	0.63 ^B^	0.75 ^B^	0.61 ^BC^	0.33 ^CD^	0.23 ^DE^	0.19 ^DE^	0.08 ^DE^	n.d. ^E^	n.d. ^E^	n.d. ^E^
1-Heptanol	972	970	0.47 ^E^	0.49 ^E^	0.43 ^E^	1.21 ^D^	0.65 ^E^	1.54 ^D^	2.75 ^C^	6.00 ^A^	5.90 ^A^	6.18 ^A^	4.32 ^B^
1-Octen-3-ol	982	978	5.34 ^A^	3.51 ^B^	1.89 ^CD^	1.49 ^CD^	1.37 ^D^	1.03 ^D^	1.55 ^CD^	1.10 ^D^	2.01 ^CD^	2.26 ^BCD^	2.67 ^BC^
***Aldehydes***													
Butanal	596	598	0.88 ^E^	0.63 ^E^	0.56 ^E^	1.70 ^D^	0.42 ^E^	0.78 ^E^	1.64 ^D^	2.97 ^C^	4.26 ^B^	5.30 ^A^	5.32 ^A^
Pentanal	700	701	26.4 ^A^	19.1 ^B^	17.9 ^B^	12.2 ^C^	11.4 ^C^	6.09 ^D^	5.34 ^DE^	3.25 ^A^	1.99 ^A^	2.29 ^F^	1.79 ^F^
2-Butenal, 2-methyl-, (*E*)-	747	737	0.16 ^H^	0.17 ^H^	0.31 ^H^	1.26 ^EF^	0.92 ^G^	1.67 ^CD^	3.73 ^A^	1.97 ^BC^	2.06 ^B^	1.44 ^DE^	1.13 ^FG^
3-Hexenal, (*Z*)	801	802	5.86 ^B^	4.37 ^C^	5.71 ^B^	4.59 ^C^	3.17 ^D^	3.81 ^CD^	4.49 ^C^	4.54 ^C^	8.08 ^A^	3.65 ^CD^	2.87 ^D^
Hexanal	803	802	107 ^A^	78.8 ^C^	96.4 ^B^	77.6 ^C^	53.3 ^D^	44.9 ^DE^	50.5 ^D^	37.2 ^EF^	24.7 ^G^	29.0 ^FG^	22.6 ^G^
2-Hexenal, (*Z*)	850	848	0.90 ^B^	0.32 ^BC^	0.84 ^BC^	0.50 ^BC^	0.19 ^C^	0.44 ^BC^	0.76 ^BC^	0.76 ^BC^	3.09 ^A^	1.01 ^B^	0.37 ^BC^
2-Hexenal, (*E*)	857	854	24.6 ^CD^	16.2 ^EF^	28.6 ^BC^	22.2 ^DE^	14.0 ^F^	24.0 ^CD^	32.4 ^B^	34.3 ^B^	81.4 ^A^	29.9 ^BC^	20.3 ^DE^
Heptanal	905	907	4.36 ^A^	2.49 ^B^	2.31 ^BC^	2.12 ^BCD^	1.30 ^CDE^	0.95 ^E^	1.14 ^DE^	0.79 ^E^	0.55 ^E^	0.65 ^E^	0.66 ^E^
2,4-Hexadienal, (*E*,*E*)-	915	913	1.99 ^A^	1.03 ^C^	1.42 ^B^	0.95 ^C^	0.54 ^E^	0.63 ^DE^	0.84 ^CD^	0.54 ^E^	2.08 ^A^	0.24 ^F^	n.d. ^F^
2-Heptenal, (*Z*)-	962	959	27.3 ^A^	17.1 ^B^	8.1 ^BC^	5.24 ^C^	5.07 ^C^	2.06 ^C^	1.90 ^C^	1.17 ^C^	0.76 ^C^	0.79 ^C^	0.52 ^C^
2-Octenal, (*E*)-	1065	1065	15.1 ^A^	8.2 ^B^	4.89 ^BC^	3.27 ^CD^	2.54 ^CD^	1.06 ^D^	1.09 ^D^	0.63 ^D^	0.37 ^D^	0.60 ^D^	0.50 ^D^
Decanal	1211	1208	1.93 ^A^	1.14 ^AB^	0.91 ^AB^	1.25 ^AB^	0.63 ^B^	0.60 ^B^	1.13 ^AB^	1.09 ^AB^	1.28 ^AB^	1.88 ^A^	1.58 ^AB^
Dodecanal	1418	1411	1.89 ^A^	0.68 ^B^	0.24 ^C^	0.28 ^C^	0.23 ^C^	0.17 ^C^	0.24 ^C^	0.27 ^C^	0.28 ^C^	0.33 ^BC^	0.28 ^C^
***Esters***													
Formic acid, methyl ester	413	401	0.41	0.54	0.42	0.47	0.37	0.41	0.41	0.35	0.37	0.39	0.32
Acetic acid, methyl ester	536	522	1.70	2.00	1.80	2.30	1.85	2.10	1.94	2.20	2.39	2.70	2.78
Acetic acid, ethyl ester	621	620	6.84 ^BCD^	7.62 ^BC^	7.77 ^B^	9.16 ^A^	7.28 ^BCD^	7.23 ^BCD^	6.67 ^CD^	6.36 ^D^	6.54 ^D^	7.04 ^BCD^	6.44 ^D^
Carbonic acid, dimethyl ester	624	620	2.21 ^AB^	2.47 ^A^	2.31 ^A^	2.43 ^A^	2.30 ^AB^	2.34 ^A^	2.21 ^AB^	2.09 ^AB^	2.12 ^AB^	2.07 ^AB^	1.90 ^B^
Propanoic acid, methyl ester	637	643	3.69 ^ABC^	4.11 ^A^	3.86 ^AB^	3.92 ^AB^	3.79 ^AB^	3.77 ^AB^	3.45 ^BCD^	3.15 ^A^	3.19 ^A^	3.08 ^DE^	2.85 ^E^
Acetic acid, propyl ester	718	720	n.d. ^F^	n.d. ^F^	0.40 ^F^	14.2 ^D^	2.31 ^F^	8.28 ^E^	14.6 ^D^	45.5 ^A^	41.2 ^A^	81.6 ^A^	66.2 ^B^
Butanoic acid, methyl est.	725	728	11.1 ^ABC^	11.9 ^A^	11.1 ^ABC^	11.3 ^AB^	11.0 ^ABC^	11.0 ^ABC^	10.2 ^BCD^	9.63 ^A^	9.82 ^A^	9.53 ^CD^	8.88 ^D^
Isobutyl acetate	777	780	0.43 ^D^	0.43 ^D^	1.16 ^D^	2.40 ^D^	0.90 ^D^	1.81 ^D^	1.30 ^D^	7.74 ^C^	9.61 ^C^	17.8 ^B^	22.4 ^A^
Butanoic acid, 3-methyl-, methyl ester	779	781	1.07 ^AB^	1.14 ^A^	1.06 ^AB^	1.03 ^ABCD^	1.03 ^ABCD^	1.03 ^ABC^	0.91 ^ABCDE^	0.84 ^CDE^	0.84 ^CDE^	0.83 ^DE^	0.77 ^E^
Butanoic acid, 2-methyl-, methyl ester	781	782	2.49 ^E^	2.70 ^DE^	2.60 ^DE^	3.19 ^ABC^	2.73 ^DE^	2.83 ^CDE^	2.97 ^CD^	3.14 ^BC^	3.40 ^AB^	3.58 ^A^	2.88 ^CDE^
Propanoic acid, propyl ester	812	811	0.02 ^E^	n.d. ^E^	0.02 ^E^	1.86 ^D^	0.32 ^E^	2.59 ^D^	2.78 ^D^	13.0 ^AB^	10.9 ^C^	14.1 ^A^	11.7 ^BC^
Acetic acid, butyl ester	816	817	6.27 ^G^	7.75 ^G^	19.6 ^G^	120.0 ^F^	30.5 ^G^	97.4 ^F^	155 ^E^	395 ^D^	482 ^C^	696 ^A^	665 ^B^
1-Butanol, 2-methyl-, acetate	882	885	20.4 ^H^	17.3 ^H^	63 ^G^	155 ^E^	124 ^F^	177 ^E^	256 ^D^	435 ^C^	457 ^C^	528 ^A^	500 ^B^
Butanoic acid, propyl est.	899	898	0.02 ^E^	n.d. ^E^	0.04 ^E^	1.29 ^D^	0.07 ^E^	0.44 ^E^	0.63 ^DE^	3.06 ^C^	4.25 ^B^	6.82 ^A^	6.60 ^A^
Propanoic acid, butyl est.	908	908	0.01 ^F^	n.d. ^F^	0.17 ^F^	5.03 ^EF^	0.86 ^F^	8.56 ^DE^	11.4 ^D^	40.5 ^C^	50.5 ^B^	62.1 ^A^	52.8 ^B^
Acetic acid, pentyl ester	914	915	5.19 ^F^	3.61 ^F^	7.51 ^EF^	14.9 ^E^	9.92 ^EF^	25.2 ^D^	34.1 ^C^	76 ^B^	83.7 ^AB^	85.8 ^A^	87.3 ^A^
2-Buten-1-ol, 3-methyl-, acetate	923	923	0.94 ^F^	0.79 ^F^	1.58 ^E^	1.97 ^DE^	2.56 ^C^	4.38 ^B^	6.71 ^A^	4.04 ^B^	2.70 ^C^	2.88 ^C^	2.36 ^CD^
Butanoic acid, 2-methyl-, propyl ester	949	938	0.04 ^G^	0.03 ^G^	0.05 ^G^	2.02 ^E^	0.15 ^FG^	0.68 ^FG^	0.82 ^F^	3.33 ^D^	4.52 ^C^	7.08 ^A^	5.42 ^B^
2-Methylpropanoic acid, butyl ester	954	n.a.	0.02 ^E^	0.01 ^E^	0.03 ^E^	0.32 ^DE^	0.03 ^E^	0.11 ^E^	0.13 ^E^	0.62 ^D^	1.56 ^C^	2.98 ^A^	2.42 ^B^
1-Butanol, 3-methyl-, propanoate	974	975	0.07 ^F^	0.06 ^F^	0.17 ^F^	1.30 ^E^	1.86 ^E^	3.54 ^D^	5.55 ^C^	7.66 ^A^	6.56 ^B^	6.6 ^B^	6.25 ^C^
Butanoic acid, butyl ester	996	996	0.24 ^E^	0.22 ^E^	0.41 ^E^	6.24 ^D^	0.51 ^E^	1.67 ^E^	2.93 ^DE^	11.0 ^C^	20.5 ^B^	30.7 ^A^	32.1 ^A^
Acetic acid, hexyl ester	1013	1013	20.5 ^G^	16.4 ^G^	28.8 ^FG^	71.8 ^E^	34.7 ^FG^	71.4 ^E^	92.2 ^D^	184 ^C^	208 ^B^	249 ^A^	250 ^A^
2-Methylbutanoic acid, butyl ester	1045	1041	0.05 ^G^	0.14 ^G^	0.78 ^G^	14.3 ^E^	1.67 ^G^	8.08 ^F^	10.4 ^EF^	27.1 ^D^	50.5 ^B^	58.3 ^A^	40.6 ^C^
2-Methylpropanoic acid, pentyl ester	1061	1056	0.09 ^E^	0.04 ^E^	0.11 ^E^	0.99 ^CD^	0.58 ^D^	0.86 ^D^	1.42 ^C^	2.37 ^B^	3.15 ^A^	3.34 ^A^	3.29 ^A^
Butanoic acid, pentyl est.	1095	1093	0.17 ^DEF^	0.03 ^F^	0.06 ^EF^	0.35 ^CD^	0.19 ^DEF^	0.27 ^CDE^	0.47 ^C^	0.92 ^B^	1.36 ^A^	1.25 ^A^	1.32 ^A^
Propanoic acid, hexyl est.	1106	1105	0.89 ^C^	0.69 ^C^	0.39 ^C^	1.68 ^C^	0.63 ^C^	2.24 ^C^	3.04 ^C^	9.96 ^B^	11.5 ^AB^	13.0 ^A^	13.8 ^A^
2-Methylbutanoic acid, 2-methylbutyl ester	1109	1104	0.1 ^G^	0.06 ^G^	0.45 ^G^	4.48 ^E^	3.35 ^F^	3.51 ^F^	7.10 ^C^	9.14 ^B^	10.3 ^A^	8.98 ^B^	6.2 ^D^
2-Methylpropanoic acid, hexyl ester	1149	1150	0.3 ^DE^	0.05 ^E^	0.05 ^E^	0.43 ^D^	0.09 ^E^	0.30 ^DE^	0.56 ^D^	1.08 ^C^	1.90 ^B^	2.27 ^A^	2.01 ^AB^
Butanoic acid, hexyl ester	1193	1191	0.26 ^D^	0.09 ^D^	0.13 ^D^	8.0 ^BC^	0.88 ^D^	1.93 ^D^	3.38 ^CD^	10.5 ^B^	17.2 ^A^	19.7 ^A^	16.7 ^A^
2-Methylbutanoic acid, hexyl ester	1241	1238	n.d. ^F^	0.16 ^F^	0.52 ^F^	26.9 ^D^	3.61 ^EF^	13.0 ^EF^	26.5 ^D^	53.1 ^C^	90.4 ^A^	76.3 ^B^	48.9 ^C^
Hexanoic acid, hexyl est.	1390	1393	1.48 ^A^	0.99 ^B^	0.52 ^EF^	0.70 ^DE^	0.39 ^F^	0.54 ^EF^	0.74 ^CDE^	0.73 ^CDE^	0.88 ^BCD^	1.01 ^B^	0.97 ^BC^
***Ketones***													
2-Butanone	601	600	21.3 ^AB^	25.8 ^A^	24.0 ^AB^	25.4 ^A^	24.0 ^AB^	24.4 ^AB^	22.9 ^AB^	21.4 ^AB^	21.5 ^AB^	21.1 ^AB^	19.1 ^B^
2-Butanone, 3-methyl-	664	666	3.36 ^ABC^	3.73 ^A^	3.48 ^AB^	3.49 ^AB^	3.41 ^AB^	3.42 ^AB^	3.12 ^BCD^	2.82 ^A^	2.83 ^A^	2.75 ^DE^	2.52 ^E^
1-Penten-3-one	689	689	3.14 ^A^	2.24 ^B^	1.27 ^C^	0.93 ^CD^	0.98 ^CD^	0.54 ^CD^	0.49 ^CD^	0.36 ^A^	0.26 ^A^	0.21 ^D^	0.19 ^D^
2-Pentanone	690	690	16.6 ^AB^	18.6 ^A^	17.9 ^AB^	18.4 ^A^	18.2 ^AB^	18.7 ^A^	17.1 ^AB^	16.1 ^A^	16.3 ^A^	15.3 ^AB^	14.7 ^B^
3-Pentanone	699	701	15.2 ^AB^	15.8 ^A^	14.5 ^ABC^	14.2 ^ABC^	14.2 ^ABC^	13.4 ^BCD^	12.4 ^CDE^	11.2 ^A^	10.6 ^A^	10.4 ^EF^	9.70 ^F^
3-Pentanone, 2-methyl-	755	752	2.81 ^ABC^	2.97 ^A^	2.75 ^ABC^	2.91 ^AB^	2.77 ^ABC^	2.79 ^ABC^	2.61 ^ABC^	2.47 ^BCD^	2.50 ^BCD^	2.38 ^CD^	2.22 ^D^
1-Octen-3-one	982	982	11.9 ^A^	8.16 ^B^	3.28 ^C^	2.25 ^C^	2.24 ^C^	1.03 ^C^	1.01 ^C^	0.80 ^C^	0.93 ^C^	1.2 ^C^	1.29 ^C^
2-Octanone	994	992	7.59 ^A^	9.25 ^A^	7.82 ^A^	8.87 ^A^	9.10 ^A^	9.17 ^A^	8.62 ^A^	8.85 ^A^	8.62 ^A^	8.57 ^A^	8.40 ^A^
5,9-Undecadien-2-one, 6,10-dimethyl-, (*E*)-	1466	1456	3.80 ^A^	1.61 ^B^	0.53 ^B^	0.50 ^B^	0.33 ^B^	0.13 ^B^	0.26 ^B^	0.14 ^B^	0.15 ^B^	0.25 ^B^	0.17 ^B^
***Others***													
Isoprene	512	503	0.96 ^EF^	1.75 ^CD^	1.64 ^D^	1.93 ^CD^	1.95 ^C^	2.75 ^B^	3.92 ^A^	1.72 ^CD^	1.12 ^E^	0.92 ^EF^	0.75 ^F^
1,3-Hexadiene, (*E*)- ^t^	631	n.a.	2.54 ^CDE^	2.32 ^CDEF^	5.25 ^A^	4.25 ^AB^	3.33 ^BC^	2.80 ^CD^	4.60 ^A^	2.51 ^CDE^	1.88 ^DEF^	1.61 ^EF^	1.38 ^F^
1-Octene	795	796	2.30 ^C^	2.55 ^BC^	2.78 ^BC^	3.48 ^ABC^	2.79 ^BC^	3.28 ^ABC^	3.59 ^AB^	3.33 ^ABC^	4.08 ^A^	3.68 ^AB^	3.51 ^ABC^
4-Octene	809	806	2.46 ^B^	2.95 ^AB^	2.77 ^AB^	3.26 ^A^	3.03 ^AB^	3.17 ^A^	3.09 ^AB^	3.05 ^AB^	3.09 ^AB^	2.86 ^AB^	2.77 ^AB^
Hexane, 1-methoxy-	830	832	2.77 ^BCD^	2.69 ^CD^	2.37 ^D^	2.84 ^ABCD^	2.39 ^D^	2.62 ^CD^	2.79 ^ABCD^	3.09 ^ABC^	3.4 ^AB^	3.45 ^A^	3.36 ^AB^
Butanoic acid, 2-methyl-	847	848	n.d. ^B^	n.d. ^B^	n.d. ^B^	4.00 ^B^	1.00 ^B^	3.70 ^B^	5.90 ^B^	10.2 ^B^	46.7 ^A^	43.0 ^A^	13.3 ^B^
Undecane	1103	1100	18.4	19.2	17.7	18.2	17.4	18.7	16.6	18.0	19.0	18.2	16.1
Dodecane	1203	1200	4.12 ^A^	2.04 ^B^	1.60 ^B^	1.69 ^B^	1.47 ^B^	1.58 ^B^	1.43 ^B^	1.62 ^B^	1.75 ^B^	1.70 ^B^	1.49 ^B^
Estragole	1215	1207	0.11 ^C^	0.09 ^C^	0.08 ^C^	3.14 ^B^	0.12 ^C^	0.85 ^C^	3.01 ^B^	5.64 ^A^	2.93 ^B^	4.79 ^A^	4.42 ^AB^
Tetradecane	1404	1400	8.26 ^A^	0.96 ^B^	0.35 ^B^	0.31 ^B^	0.20 ^B^	0.18 ^B^	0.17 ^B^	0.21 ^B^	0.18 ^B^	0.19 ^B^	0.17 ^B^
β-Damascenone, (*E*)-	1406	1396	3.60	3.50	3.60	3.10	2.10	2.20	2.24	1.60	1.61	1.93	1.78

^a^ The identification was based on agreement between mass spectra of the analyzed compounds with those from MS libraries and LRI corresponding to those from databases (in-house database built with authentic reference compounds; www.flavornet.org; www.odour.or.uk; Nist Webbook; accessed on 5 August 2022). A maximum of 10 RI units between data from the literature and the experimental data was excepted in cases when the authentic reference compound was not available. ^b^ Linear temperature programmed retention index determined on a DB5 column. ^c^ Linear temperature programmed retention index from databases (in-house database built with authentic reference compounds; www.flavornet.org; www.odour.or.uk; Nist Webbook; accessed on 23 March 2022). ^d^ Relative concentration (mean, *n* = 2) collected from the headspace using HS-SPME calculated by comparison of the peak areas with that of the internal standard 2-octanol (30 ng absolute) with a response factor of 1. One-way ANOVA followed by post hoc pairwise comparison using Tukey’s Honestly Significant Difference (HSD) test control (*p* < 0.05) to check for differences between single samples. ^t^ Compounds with a match in the MS libraries but without RI in the databases (n.a.) were tentatively identified. ^A,B,C,D,E,F,G,H,I,^ different superscript letters indicate statistically significant differences (*p* < 0.05) among the Crimson Crisp apples from all harvests.

**Table 3:** Relative concentrations (average values (*n* = 2); µg/L) of the 2° aroma compounds identified in apple juice produced from Crimson Crisp apples harvested at different stages of maturity (104–188 dafb).

Compound Name ^a^	RI [DB5] (exp.) ^b^	RI [DB5] (lit.) ^c^	104 dafb ^d^ [µg/L]	111 dafb ^d^ [µg/L]	118 dafb ^d^ [µg/L]	125 dafb ^d^ [µg/L]	132 dafb ^d^ [µg/L]	139 dafb ^d^ [µg/L]	146 dafb ^d^ [µg/L]	153 dafb ^d^ [µg/L]	160 dafb ^d^ [µg/L]	174 dafb ^d^ [µg/L]	188 dafb ^d^ [µg/L]
***Alcohols***													
Ethanol	475	477	2.26	2.38	2.36	2.89	2.55	2.54	2.39	2.49	2.40	2.62	2.94
2-Propanol	508	506	0.84 ^B^	0.70 ^B^	0.92 ^B^	0.81 ^B^	0.71 ^B^	0.81 ^B^	0.73 ^B^	0.97 ^B^	0.92 ^B^	1.05 ^B^	1.70 ^A^
2-Propanol, 2-methyl-	534	530	0.11 ^AB^	0.10 ^AB^	0.07 ^AB^	0.09 ^AB^	0.13 ^A^	0.11 ^AB^	0.10 ^AB^	0.08 ^AB^	0.07 ^B^	0.07 ^AB^	0.07 ^AB^
1-Propanol	566	558	0.82 ^F^	0.94 ^F^	1.14 ^F^	17.0 ^CD^	1.67 ^F^	5.15 ^EF^	11.0 ^DE^	24.95 ^B^	23.53 ^BC^	35.9 ^A^	35.79 ^A^
2-Butanol	607	609	0.48 ^E^	0.57 ^DE^	0.69 ^CDE^	0.98 ^BC^	0.75 ^CDE^	0.77 ^CDE^	0.91 ^BCD^	0.73 ^CDE^	0.95 ^BC^	1.08 ^B^	1.56 ^A^
3-Buten-2-ol, 2-methyl-	615	614	1.63	2.26	2.38	2.06	2.53	2.20	2.55	1.89	2.00	1.61	1.90
1-Propanol, 2-methyl-	632	635	0.76 ^G^	0.99 ^G^	1.99 ^G^	9.53 ^EF^	2.66 ^G^	5.99 ^FG^	12.2 ^DE^	16.6 ^CD^	20.6 ^BC^	25.0 ^B^	37.8 ^A^
1-Butanol	668	670	7.80 ^F^	9.46 ^F^	23.7 ^F^	356 ^D^	34.5 ^EF^	165 ^E^	371 ^D^	609 ^C^	773 ^B^	892 ^A^	883 ^A^
2-Propanol, 1-methoxy-	685	672	15.9	10.4	12.0	12.9	8.30	16.84	9.70	80,0	18.6	12.8	12.3
3-Buten-1-ol, 3-methyl- ^t^	732	n.a.	0.58 ^E^	0.93 ^CDE^	0.71 ^DE^	0.99 ^CDE^	0.99 ^CDE^	1.16 ^BCD^	1.32 ^ABC^	1.54 ^A^	1.50 ^A^	1.35 ^AB^	1.23 ^ABC^
1-Butanol, 3-methyl-	735	738	1.23 ^BCD^	1.55 ^ABCD^	0.98 ^CD^	1.78 ^AB^	0.95 ^D^	0.96 ^D^	1.87 ^AB^	1.70 ^ABC^	1.92 ^A^	1.74 ^AB^	1.75 ^AB^
1-Butanol, 2-methyl-	740	738	15.3 ^E^	19.6 ^E^	70.2 ^E^	392	120.2 ^E^	278 ^D^	567 ^B^	670 ^A^	703 ^A^	621 ^AB^	589 ^AB^
1-Pentanol	767	771	2.23 ^F^	3.21 ^F^	6.36 ^F^	26.6 ^DE^	11.7 ^EF^	36.5 ^D^	83.9 ^C^	102.6 ^B^	121.9 ^A^	89.5 ^BC^	70.2 ^C^
2-Buten-1-ol, 3-methyl-	774	772	2.35 ^F^	3.39 ^EF^	4.87 ^EF^	10.2 ^CD^	14.7 ^BC^	16.9 ^B^	33.0 ^A^	15.6 ^B^	10.4 ^CD^	6.95 ^DE^	5.73 ^DEF^
3-Hexen-1-ol, (*Z*)-	857	852	4.30 ^E^	5.17 ^E^	5.69 ^DE^	4.46 ^E^	8.74 ^C^	13.2 ^B^	4.60 ^E^	15.7 ^A^	11.8 ^B^	7.49 ^CD^	3.45 ^E^
2-Hexen-1-ol, (*E*)-	866	867	55.0 ^CDEF^	76.2 ^B^	44.0 ^EF^	106 ^A^	50.5 ^DEF^	68.4 ^BCD^	73.6 ^BC^	75.1 ^B^	62.5 ^BCDE^	60.4 ^BCDE^	40.2 ^F^
1-Hexanol	870	870	37.9 ^F^	80.9 ^F^	42.5 ^F^	306 ^D^	59.6 ^F^	202 ^E^	389 ^D^	597 ^C^	737 ^A^	690 ^AB^	610 ^BC^
2-Propanol, 1-butoxy-	942	947	0.59 ^CD^	0.83 ^ABCD^	0.52 ^D^	0.96 ^AB^	0.85 ^ABCD^	1.14 ^A^	0.99 ^AB^	0.76 ^ABCD^	0.68 ^BCD^	0.88 ^ABC^	0.81 ^ABCD^
1-Heptanol	969	970	0.46 ^E^	0.50 ^E^	0.57 ^E^	2.04 ^D^	0.69 ^E^	1.90 ^D^	3.59 ^C^	6.23 ^AB^	6.74 ^A^	6.74 ^A^	5.26 ^B^
1-Octen-3-ol	979	978	0.37 ^F^	0.40 ^EF^	0.42 ^EF^	0.63 ^EF^	0.50 ^EF^	0.72 ^E^	1.06 ^D^	1.20 ^D^	2.00 ^C^	3.30 ^A^	2.44 ^B^
5-Hepten-2-ol, 6-methyl-	993	993	0.66 ^DE^	0.70 ^DE^	0.64 ^DE^	0.80 ^DE^	0.65 ^E^	0.71 ^DE^	0.86 ^DE^	0.96 ^D^	1.45 ^C^	2.62 ^B^	2.98 ^A^
1-Hexanol, 2-ethyl-	1030	1031	4.20 ^D^	3.85 ^D^	3.31 ^D^	4.64 ^D^	3.38 ^D^	4.09 ^D^	3.74 ^D^	3.05 ^C^	3.53 ^B^	3.79 ^A^	3.40 ^B^
1-Octanol	1070	1070	4.25 ^F^	4.30 ^F^	4.09 ^F^	7.49 ^D^	4.58 ^F^	5.54 ^EF^	6.75 ^DE^	10.05 ^C^	12.6 ^A^	16.4 ^A^	14.4 ^B^
1-Decanol	1274	1272	0.73 ^F^	0.62 ^F^	0.44 ^F^	0.68 ^DE^	0.6 ^F^	0.71 ^EF^	0.80 ^D^	0.82 ^C^	1.01 ^B^	1.77 ^A^	1.88 ^B^
***Aldehydes***													
Propanal	499	498	0.26 ^DE^	0.25 ^DE^	0.27 ^DE^	0.34 ^CDE^	0.26 ^DE^	0.24 ^E^	0.26 ^DE^	0.41 ^CD^	0.50 ^BC^	0.68 ^A^	0.61 ^AB^
Butanal	594	598	9.3 ^A^	9.23 ^A^	7.19 ^AB^	5.19 ^BC^	4.13 ^C^	3.05 ^C^	3.43 ^C^	3.41 ^C^	4.77 ^C^	5.00 ^BC^	4.81 ^BC^
2-Pentenal, (*E*)-	745	744	0.23 ^E^	0.28 ^E^	0.38 ^E^	1.06 ^CD^	0.95 ^D^	1.13 ^CD^	2.34 ^A^	1.66 ^B^	1.69 ^B^	1.39 ^BC^	0.85 ^D^
3-Hexenal, (*Z*)-	799	802	30.1 ^AB^	31.9 ^AB^	33.2 ^A^	25.8 ^BC^	31.5 ^AB^	21.1 ^CDE^	27.6 ^BC^	23.0 ^CD^	19.4 ^CDE^	14.1 ^E^	17.1 ^DE^
Hexanal	800	802	564 ^A^	552 ^A^	412 ^B^	301 ^C^	236 ^CD^	174 ^DE^	240 ^CDE^	202 ^DE^	150 ^E^	175 ^DE^	182 ^DE^
2-Hexenal, (*Z*)-	847	848	25.2 ^AB^	27.1 ^A^	24.6 ^AB^	18.5 ^CDE^	21.0 ^ABC^	13.1 ^EF^	20.4 ^BCD^	15.8 ^CDEF^	10.8 ^F^	9.30 ^F^	12.9 ^DEF^
2-Hexenal, (*E*)-	856	855	508 ^AB^	537 ^A^	490 ^AB^	425 ^BC^	451 ^BC^	344 ^DE^	456 ^BC^	406 ^CD^	357 ^CDE^	299 ^E^	347 ^CDE^
2,4-Hexadienal, (*E*,*E*)-	912	913	26.0 ^A^	26.5 ^A^	23.7 ^AB^	18.8 ^CD^	20.8 ^BC^	14.6 ^DEF^	19.5 ^CD^	16.2 ^CDE^	13.3 ^EF^	10.58 ^F^	13.22 ^DEF^
Octanal	1004	1005	1.7	1.5	1.4	2.4	2.2	2.4	2.1	3.1	3.9	5.1	3.0
Nonanal	1106	1106	8.40 ^F^	7.68 ^F^	7.24 ^F^	13.3 ^D^	10.3 ^EF^	9.90 ^DE^	11.53 ^C^	10.4 ^A^	12.9 ^A^	20.0 ^A^	10.8 ^B^
Decanal	1209	1208	3.49 ^F^	2.44 ^F^	2.04 ^F^	3.98 ^E^	2.81 ^F^	3.33 ^EF^	3.6 ^E^	3.31 ^D^	3.7 ^C^	7.69 ^A^	3.23 ^B^
***Esters***													
Acetic acid, methyl ester	535	525	0.50 ^C^	0.50 ^C^	0.51 ^C^	0.58 ^BC^	0.54 ^C^	0.51 ^C^	0.56 ^C^	0.66 ^BC^	0.77 ^ABC^	0.88 ^AB^	1.00 ^A^
Acetic acid, ethyl ester	619	620	2.34	2.50	2.78	2.72	2.76	2.50	2.31	2.27	2.41	2.58	2.90
Carbonic acid, dimethyl ester	622	620	1.88	1.93	1.98	1.87	2.02	1.89	1.78	1.74	1.72	1.69	1.81
Propanoic acid, methyl ester	635	630	1.22	1.27	1.36	1.21	1.35	1.24	1.14	1.09	1.05	1.05	1.13
Acetic acid, propyl ester	715	720	0.01 ^D^	0.03 ^D^	0.32 ^D^	10.2 ^C^	0.52 ^D^	2.74 ^D^	6.76 ^CD^	18.9 ^B^	25.5 ^B^	44.9 ^A^	40.3 ^A^
Butanoic acid, methyl ester	724	728	3.61 ^ABC^	3.63 ^ABC^	4.03 ^A^	3.43 ^ABC^	4.1 ^AB^	3.85 ^ABC^	3.3 ^ABC^	2.98 ^ABC^	2.78 ^BC^	2.63 ^C^	2.98 ^ABC^
Acetic acid, 2-methylpropyl ester	774	780	0.30 ^E^	0.40 ^E^	0.97 ^E^	1.38 ^E^	0.40 ^E^	1.03 ^E^	1.53 ^E^	3.59 ^D^	6.77 ^C^	9.43 ^B^	12.4 ^A^
2-Methylbutanoic acid, methyl ester	779	782	1.85 ^B^	2.00 ^AB^	1.98 ^AB^	2.35 ^AB^	2.04 ^AB^	2.07 ^AB^	1.83 ^B^	2.25 ^AB^	2.61 ^A^	2.49 ^A^	1.85 ^AB^
Propanoic acid, propyl est.	810	811	0.12 ^D^	0.12 ^D^	0.16 ^D^	1.56 ^D^	0.22 ^D^	1.06 ^D^	1.65 ^D^	6.32 ^C^	8.12 ^B^	10.7 ^A^	8.23 ^B^
Acetic acid, butyl ester	813	817	5.62 ^F^	6.92 ^F^	17.1 ^F^	115 ^DE^	12.9 ^F^	60.3 ^EF^	137 ^D^	293 ^C^	475 ^B^	591 ^A^	554 ^A^
1-Butanol, 2-methyl-, acetate	878	885	13.4 ^E^	16.2 ^E^	64.1 ^DE^	114 ^D^	55.3 ^DE^	109 ^D^	195 ^C^	310 ^B^	414 ^A^	413 ^A^	364 ^A^
Butanoic acid, propyl ester	897	898	n.d. ^E^	n.d. ^E^	0.02 ^E^	0.74 ^E^	0.04 ^E^	0.19 ^E^	0.39 ^E^	1.82 ^D^	3.86 ^C^	6.12 ^A^	4.80 ^B^
Propanoic acid, butyl ester	907	908	0.01 ^D^	0.04 ^D^	0.14 ^D^	3.28 ^D^	0.44 ^D^	3.85 ^D^	6.42 ^D^	21.2 ^C^	40.2 ^B^	46.2 ^A^	35.2 ^B^
Acetic acid, pentyl ester	911	915	3.95 ^E^	4.37 ^DE^	6.8 ^DE^	10.4 ^DE^	7.05 ^DE^	16.1 ^D^	29.3 ^C^	49.0 ^B^	80.5 ^A^	71.0 ^A^	52.2 ^B^
Acetic acid, 3-methyl-2-butenyl ester	920	923	0.76 ^E^	1.12 ^DE^	1.60 ^CD^	1.62 ^D^	2.81 ^B^	2.89 ^B^	4.61 ^A^	2.88 ^B^	2.64 ^B^	2.25 ^BC^	1.71 ^CD^
Hexanoic acid, methyl est.	923	923	1.34 ^A^	1.26 ^A^	0.94 ^B^	0.81 ^BC^	0.70 ^CD^	0.56 ^D^	0.65 ^CD^	0.74 ^CD^	0.68 ^CD^	0.76 ^BC^	0.65 ^CD^
2-Methylbutanoic acid, propyl ester	946	938	n.d. ^D^	n.d. ^D^	0.02 ^D^	1.24 ^C^	0.04 ^D^	0.21 ^D^	0.41 ^D^	1.69 ^C^	3.02 ^B^	3.97 ^A^	2.8 ^B^
Propanoic acid, pentyl est.	970	972	0.24 ^G^	0.23 ^G^	0.44 ^G^	1.19 ^FG^	1.56 ^EF^	2.38 ^E^	3.54 ^D^	5.18 ^BC^	6.14 ^A^	5.79 ^AB^	4.58 ^C^
Butanoic acid, butyl ester	994	996	4.22 ^D^	4.11 ^D^	4.12 ^D^	7.04 ^D^	4.62 ^D^	4.91 ^D^	6.40 ^D^	11.4 ^C^	21.1 ^B^	30.9 ^A^	25.8 ^A^
Acetic acid, hexyl ester	1010	1013	3.38 ^B^	3.86 ^A^	4.59 ^B^	13.2 ^A^	4.57 ^A^	10.5 ^A^	17.9 ^B^	44.5 ^A^	95.4 ^A^	111 ^A^	86.7 ^B^
2-Hexen-1-ol, acetate, (*Z*)-	1012	1007	4.00 ^BC^	8.03 ^C^	3.65 ^C^	9.30 ^BC^	7.89 ^BC^	10.1 ^BC^	4.18 ^BC^	8.81 ^ABC^	9.62 ^AB^	8.36 ^A^	3.28 ^ABC^
2-Methylbutanoic acid, butyl ester	1042	1041	0.16	0.18	0.5	6.07	0.46	2.44	4.65	12.3	27.7	29.6	19.2
Propanoic acid, hexyl ester	1102	1105	0.11 ^BCD^	0.10 ^BC^	0.10 ^CD^	0.51 ^A^	0.17 ^BCD^	0.6 ^CDE^	0.98 ^E^	2.86 ^DE^	4.49 ^B^	5.44 ^A^	4.5 ^A^
2-Methylbutanoic acid, 2-methylbutyl ester	1105	1104	0.12	0.10	0.22	1.16	0.41	0.79	1.72	2.84	3.06	3.02	2.11
Octanoic acid, methyl est.	1123	1120	0.14 ^B^	0.12 ^B^	0.65 ^B^	0.47 ^AB^	0.66 ^B^	3.80 ^B^	0.43 ^B^	1.04 ^B^	0.35 ^AB^	0.92 ^A^	4.20 ^B^
3-(Methylthio)propyl acetate	1126	1123	0.07	0.12	0.19	0.30	0.16	0.62	0.49	0.84	1.12	2.08	2.76
Butanoic acid, hexyl ester	1191	1191	0.10 ^G^	0.11 ^G^	0.09 ^FG^	0.93 ^EFG^	0.19 ^G^	0.39 ^DE^	0.87 ^EF^	1.95 ^CD^	3.06 ^C^	5.34 ^B^	3.51 ^A^
2-Methylbutanoic acid, hexyl ester	1238	1238	0.30 ^B^	0.34 ^B^	0.35 ^B^	3.20 ^B^	0.55 ^B^	1.80 ^B^	4.57 ^B^	8.54 ^B^	12.0 ^B^	16.1 ^A^	10.1 ^B^
2-Methylpropanoic acid, 2-ethyl-3-hydroxyhexyl ester	1391	1373	1.01 ^A^	0.92 ^AB^	0.70 ^ABC^	0.92 ^ABC^	1.32 ^ABC^	1.14 ^ABC^	0.92 ^BC^	0.92 ^C^	0.97 ^BC^	1.04 ^ABC^	0.96 ^ABC^
***Ketones***													
2-Butanone	599	600	12.0	12.2	12.9	11.5	13.1	12.1	11.0	10.7	10.4	10.2	11.4
2-Butanone, 3-methyl	662	666	0.65	0.63	0.66	0.41	0.41	0.60	0.37	0.30	0.36	0.37	0.56
2-Pentanone	689	690	9.82	10.0	10.6	9.7	10.8	9.95	9.36	8.77	8.43	8.47	9.12
3-Pentanone	698	701	8.86 ^AB^	8.93 ^AB^	9.67 ^A^	8.36 ^AB^	9.64 ^AB^	8.77 ^AB^	7.67 ^AB^	7.00 ^B^	6.90 ^B^	6.95 ^AB^	7.23 ^AB^
3-Pentanone, 2-methyl-	753	752	1.20	1.22	1.33	1.19	1.30	1.22	1.15	1.07	1.04	1.06	1.15
1-Octen-3-one	979	982	0.31 ^F^	0.27 ^F^	0.24 ^F^	0.7 ^EF^	0.32 ^F^	0.46 ^EF^	0.93 ^E^	1.52 ^D^	2.44 ^C^	4.79 ^B^	5.81 ^A^
2-Octanone	992	992	8.34	8.27	8.45	7.61	8.33	7.92	7.61	7.49	6.97	6.91	7.40
***Others***													
Butanoic acid, 2-methyl-	846	848	0.19 ^C^	0.18 ^C^	0.13 ^C^	9.31 ^C^	3.22 ^C^	7.34 ^C^	8.0 ^C^	44.2 ^B^	74.6 ^A^	71.5 ^A^	36.0 ^B^
α-Pinene	946	939	0.32	0.18	0.14	0.15	0.31	0.15	0.35	0.15	0.20	0.11	0.16
Hexanal dimethyl acetal ^t^	977	n.a.	8.98 ^A^	8.55 ^A^	5.83 ^B^	4.67 ^BC^	3.27 ^CD^	2.42 ^D^	3.47 ^CD^	3.53 ^CD^	2.63 ^CD^	3.34 ^CD^	3.14 ^CD^
Linalool oxide	1084	1088	0.83 ^F^	0.89 ^F^	0.81 ^F^	1.44 ^D^	0.83 ^EF^	0.74 ^EF^	0.62 ^DE^	0.69 ^C^	0.92 ^B^	1.26 ^A^	1.2 ^A^
Undecane	1101	1100	13.4 ^E^	13.0 ^E^	13.0 ^E^	12.5 ^DE^	12.9 ^E^	11.9 ^DE^	12.3 ^D^	11.3 ^C^	11.0 ^B^	11.0 ^A^	11.5 ^B^
β-Damascenone, (*E*)-	1406	1396	1.95	2.18	2.09	1.75	1.68	1.80	1.20	0.89	1.04	0.99	0.70

^a^ The identification was based on agreement between mass spectra of the analyzed compounds with those from MS libraries and LRI corresponding to those from databases (in-house database built with authentic reference compounds; www.flavornet.org; www.odour.or.uk; Nist Webbook, accessed on 5 August 2022). A maximum of 10 RI units between data from the literature and the experimental data was excepted in cases when the authentic reference compound was not available. ^b^ Linear temperature programmed retention index determined on a DB5 column. ^c^ Linear temperature programmed retention index from databases (www.flavornet.org; www.odour.or.uk; Nist Webbook). ^d^ Relative concentration (mean, n = 2) collected from the headspace using HS-SPME calculated by comparison of the peak areas with that of the internal standard. 2-octanol (30 ng absolute) with a response factor of 1. One-way ANOVA followed by post hoc pairwise comparison using Tukey’s honestly significant difference (HSD) test control (*p* < 0.05) to check for differences between single samples. ^t^ Compounds with a match in the MS libraries but without reported linear retention indices in the databases were tentatively identified. ^A,B,C,D,E,F,G^ different superscript letters indicate statistically significant differences (*p* < 0.05) among the Crimson Crisp apples from all harvests.

## Data Availability

The data presented in this study are available on request from the corresponding author.
